# Interrater agreement in classifying infections during extracorporeal membrane oxygenation

**DOI:** 10.1177/03913988231193448

**Published:** 2023-08-19

**Authors:** Karlijn Verkerk, Lara CA Pladet, Christiaan L Meuwese, Dirk W Donker, Lennie PG Derde, Olaf L Cremer

**Affiliations:** 1 Department of Intensive Care Medicine, University Medical Center Utrecht, Utrecht, The Netherlands; 2 Department of Cardiology, Thoraxcenter, Erasmus, Rotterdam, The Netherlands; 3 Department of Intensive Care, Erasmus Medical Center Rotterdam, Rotterdam, The Netherlands; 4 Cardiovascular and Respiratory Physiology, TechMed Center, University of Twente, Enschede, The Netherlands

**Keywords:** Extracorporeal membrane oxygenation, extra corporeal life support, infections, interrater agreement, ECMO, ECLS

## Abstract

Infectious complications are common during extracorporeal membrane oxygenation (ECMO) and may negatively impact outcomes. However, there is considerable variation in the reported rates of incidence, which hampers the use of infections as a quality benchmark for ECMO centers. To assess the contributing role of poor interrater agreement, three independent raters reviewed medical records from all intensive care unit (ICU) patients who received ECMO for >24 h in our tertiary center between October 2019 and October 2021 for suspected episodes of infection, which were rated based on their date of onset and presumed site/diagnosis. To establish a gold standard, any discrepancies were resolved using an expert panel consisting of two intensivists/infectious disease specialists. During 83 ECMO-runs in 77 patients, we observed a total of 62 adjudicated infectious episodes (incidence rate 62, 95% CI: 48–80, per 1000 days at risk). Among 81 episodes suspected by at least one observer, 66 (81%) were identified by two, and only 44 (54%) by all three raters, resulting in Fleiss’ kappa of 0.10 (95% CI: 0.00–0.19; slight agreement). However, if raters concurred regarding infection onset, subsequent agreement on infection site was good (concordance 89%; kappa 0.85, 95% CI: 0.72–0.98; near perfect agreement). In conclusion, adjudication of infectious episodes during ECMO is associated with poor interrater agreement regarding occurrence—but not site—of infection. This finding might partially explain the significant disparities observed in reported infection rates during ECMO, emphasizing the need for caution when interpreting infection data in this particular population due to the potential for inherent measurement error.

## Introduction

Extracorporeal membrane oxygenation (ECMO) is used to provide circulatory and respiratory support in patients with severe hemodynamic shock and/or respiratory failure. ECMO is a highly invasive support modality reserved to specialized intensive care unit (ICU) settings and is associated with a wide range of circulatory complications, including thromboembolism, coagulopathy, major bleeding, limb ischemia, and circuit failure.^1^ In addition, nosocomial infections are among the most frequently encountered complications during ECMO-treatment.^2^ However, there is substantial variability in the reported occurrence of ECMO-related infections (ERIs), with incidence rates varying between 12 and 75 episodes per 1000 days at risk, and cumulative incidences between 9% and 65%.^3–5^ Furthermore, there are significant inconsistencies between studies regarding the reported association between ERIs and adverse patient outcomes (e.g. increased mortality and prolonged ICU length of stay).^3–5^ Despite these uncertainties, ERI occurrence is currently included as a quality benchmark for ECMO-centers, for example by the Extracorporeal Life Support Organization (ELSO) registry.^6^ For these reasons, it is important to explore methodological factors that could affect the robustness of this criterion.

In the general ICU-population, and ECMO-patients in particular, parameters used for diagnosing infections are difficult to interpret. Whereas non-infectious etiologies of systemic inflammation are already quite common in critically ill patients in general,^7^ ECMO-support invariably presents a further inflammatory trigger due to blood contact with non-endothelialized surfaces of the circuit itself.^2^ At the same time, fever in these patients may be masked due to effects of the heater-cooler, and radiological signs of infection may be obscured by the presence of concomitant cardiogenic pulmonary edema or the use of extreme lung-protective ventilator strategies causing airspace opacification.^2^ For these reasons we suspected the precision in diagnosing infections in an ECMO population to be poor, which would explain the variability in reported ERI occurrence at least in part. To test this hypothesis, we performed an observational study to assess true ERI incidence as well as evaluate its inherent measurement error due to interrater variability.

## Methods

This study was conducted in the mixed ICU of the University Medical Center Utrecht, The Netherlands. Study patients had been previously enrolled in the Prediction of Weanability, Survival, and Functional Outcomes after ECLS (PRECISE-ECLS) cohort (NCT05444764) as well as in the Molecular Diagnosis and Risk Stratification of Sepsis (MARS) biorepository (NCT01905033). The institutional medical ethics committee approved an opt-out method of informed consent for both studies (reference numbers 21-604/C and 10-056). Consecutive adults who had received venoarterial or venovenous ECMO-support for >24 h between October 2019 and October 2021 were selected for inclusion. There were no exclusion criteria.

ERI was defined as any new occurrence of a clinically suspected or microbiologically proven infection for which systemic antimicrobial therapy had been initiated in the period ranging from 24 h after initiation up until discontinuation of ECMO-support. Of note, all patients received selective digestive decontamination which included 4–days use of a third-generation cephalosporin. This as well as any other prophylactic or pre-emptive usage of antimicrobials was disregarded for ERI assessment.

Patient characteristics, details on the ECMO-procedure and clinical outcomes were collected from the PRECISE-ECLS database. As part of the MARS project, all suspected infectious episodes had already been prospectively adjudicated once by review of medical records by a single observer using strict definitions (further denoted as rater A).^8^ For the current analysis, two additional reviewers (raters B and C) independently performed a blinded reassessment of infection status for each day on ECMO-support. To this end, medical records as well as radiology and microbiology findings were examined in detail. This yielded three lists of suspected infectious events for each patient, including their date of onset and presumed site.

To establish an unbiased estimate of ERI incidence we used a “gold standard” reference diagnosis. A diagnosed infectious episode was considered true if all three observers had independently concurred on both infection onset and site. If this condition was not met, a consensus diagnosis was made by an expert panel consisting of two experienced intensivists with expertise in the field of ICU-acquired infections (OC and LD).

Agreement between the three raters was first assessed with respect to ERI presence or absence. During this analysis, a 48-h margin of error regarding estimated infection onset date was allowed. For example, two raters would be in agreement if they had dated infection onset on Monday and Tuesday, respectively, yet would disagree if this were Monday and Thursday (as this would likely constitute an entirely different event). Subsequently, for universally recognized ERIs only, agreement on the primary site of infection was assessed across three categories (bloodstream, pulmonary, or other infectious focus). Interrater agreement was expressed as observed concordance (%) and Cohen’s kappa (κ) using Fleiss’ generalization for multiple raters.^9^ The interpretation of κ was in accordance with Landis and Koch.^10^ Additionally, each rater’s concordance with the “*gold standard*” was determined. All analyses were performed in R version 4.0.3.

## Results

During 83 ECMO-runs in 77 patients, we observed 62 infections according to the reference diagnosis (30 bloodstream infections, 30 cases of ventilator associated pneumonia, 1 abdominal infection, and 1 mediastinitis). Overall, 40 (48%) runs were complicated by at least a single infection (Table A1). The ERI incidence rate was 62 (95% CI: 48–80) events per 1000 ECMO support days.

**Figure 1.**
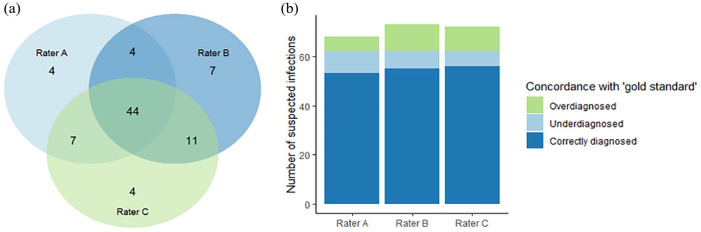
(a) Venn diagram showing discrepancies in infection classification by individual raters. Overall, 81 infectious episodes were suspected by ⩾1 rater, 66 by ⩾2 raters, and 44 by all 3 raters. (b) Bar chart showing concordance between infections classified by individual raters and the reference diagnosis.

Among 81 episodes suspected by at least a single observer, 66 (81%) were identified by two, and only 44 (54%) by all three raters, resulting in a kappa of 0.10 (95% CI: 0.00–0.19); slight agreement (Figure 1(a)). The observed concordance between raters and the expert panel is shown in Figure 1(b). For individual observers, the rate of under- and overdiagnosis infections varied between 9.7%–14.5% and 9.7%–17.7%, respectively. Fleiss’ κ for agreement on the presence or absence of infection across the three raters was 0.10 (95% CI: 0.00–0.19), indicating only slight agreement. However, in the 44 cases that were universally observed by all three raters, agreement on the presumed site of infections was good (concordance 38/44 (89%); Fleiss’ κ 0.85 (95% CI: 0.72–0.98), indicating near-perfect agreement).

## Discussion

To our knowledge, this is the first study to systematically evaluate interrater agreement for the identification of nosocomial infections in patients receiving ECMO-support. Our findings confirm the great diagnostic challenges we had anticipated in this population due to its specific characteristics, including omnipresent systemic inflammation, disrupted thermoregulation, and difficult-to-interpret chest radiographs.^2,4^ A post-hoc qualitative assessment of the 37 discordant cases that had been reviewed by the expert panel revealed that patients who had consistently elevated inflammation markers with only minor fluctuations offered the greatest diagnostic challenge. However, once raters agreed on infection onset, there was good subsequent concordance on the diagnosis (i.e. presumed site of infection), which concurs with previous observations made in a general ICU popuation.^8^

Apart from differences in population characteristics, the large variability in reported incidence rates may stem from a strong reliance of ERI diagnosis on local hospital protocols (including the extent of microbiological surveillance) and the definitions used to classify infections.^3^ For example, most prior studies as well as the ELSO registry report only culture-proven infections,^3–5^ whereas in our study we deliberately chose to include also clinically suspected infections, as not all clinically relevant infections in ICU patients can be documented with microbiological evidence. This comprehensive definition probably increased apparent ERI incidence rates (i.e. 62 infections per 1000 days at risk in our study versus a range of 12–75 reported in literature^3–5^) and may have introduced some subjectivity. However, a restricted focus on culture-proven infections only represents an oversimplification of the diagnostic complexities encountered in an ECMO-population and would have created a significant underestimation of real-world ERI incidence.

Although risk stratification was not a primary aim of this study, we observed several patient- and circuit-specific factors that were associated with an increased ERI occurrence, including longer hospitalization prior to ECMO initiation and veno-venous configuration (Table A1). None of these seem to be directly modifiable, yet some could potentially be used to inform preventive measures and/or antimicrobial treatment in ECMO patients.

Our study has certain limitations, such as a potential lack of generalizability due to specific ERI definitions used by us and variability in local diagnostic practices. For instance, despite it being often debated in literature, the use of selected decontamination of the digestive tract in mechanically ventilated patients has become standard of care in the Netherlands.^11,12^ This likely contributed to lower rates of Gram-negative pulmonary infections in this cohort.^11^ However, challenges regarding the correct identification of infections are universal and we feel that our diagnostic protocols are in line with common clinical practice. Unfortunately, due to limited sample size, we were unable to perform subgroup analyses into specific factors that may have contributed to the observed poor interrater agreement.

In conclusion, diagnostic adjudication in ECMO patients is associated with poor interrater agreement regarding the occurrence—but not site—of infection. The resulting diagnostic error warrants caution when interpreting infection epidemiology data in this population, precluding their use as a performance benchmark.

## Appendix

### Appendix A

**Table A1.** Patient characteristics upon ECMO initiation.

Characteristic	⩾1 infection (*n* = 40)	No infection (*n* = 43)	*p*-Value
Age	53 [47–61]	56 [50–64]	0.34
Sex (male)	30 (75)	25 (58)	0.16
Body mass index	25 [23–28]	27 [23–30]	0.48
Clinical frailty scale score	2 [2–4]	3 [2–5]	0.13
Comorbidities			
Diabetes mellitus	4 (10)	8 (19)	0.35
Immunocompromised status	6 (21)	4 (13)	0.49
Sequential organ failure assessment score	9 [7–11]	9 [7–10]	0.78
Prior days in the hospital	7 [2–16]	1 [0–4]	<0.01
Prior infection	12 (30)	6 (14)	0.11
Primary ECMO configuration			<0.01
Veno-arterial	13 (33)	24 (56)	
Veno-venous	26 (65)	14 (33)	
Other/combined mode	1 (3)	5 (12)	
Mode of cannulation^a^			0.56
Percutaneous	32 (80)	36 (84)	
Surgical	8 (20)	6 (14)	
ECMO indication			0.11
Circulatory arrest^b^	2 (5)	6 (14)	
Myocardial infarction	4 (10)	4 (9)	
Post-cardiotomy cardiogenic shock^c^	6 (15)	6 (14)	
ARDS	12 (30)	6 (14)	
Lung transplantation^d^	3 (8)	11 (26)	
Other	13 (38)	10 (23)	
ECMO duration (days)	13 [8–21]	5 [3–9]	<0.01
Mechanical ventilation duration (days)	25 [19–39]	15 [4–25]	<0.01
ICU length-of-stay (days)	31 [21–41]	19 [10–32]	<0.01
Hospital length-of-stay^e^ (days)	51 [26–75]	36 [21–54]	0.04
In-hospital mortality	15 (38)	10 (23)	0.24

Characteristics reflect patient condition at the start of ECMO support (six patients had multiple runs and are represented twice). Data show absolute counts (%) for categorical variables and medians [interquartile range] for continuous variables.

a Type of cannulation unknown for one patient.

b Includes pulmonary embolism, dysrhythmia, intoxication, and other indications for extracorporeal cardiopulmonary resuscitation.

c Includes left- and right ventricular failure.

d Includes primary graft dysfunction and pulmonary hypertension.

e Regardless of outcome.
